# Supplementary material for: Identification of photoperiod-induced specific miRNAs in the adrenal glands of Sunite sheep (Ovis aries)
Source: Front Vet Sci. 2022 Jul 22;9:888207. doi: 10.3389/fvets.2022.888207 (PMC9354845; doi:10.3389/fvets.2022.888207)
Supplement: Supplementary file 1 [file Data_Sheet_1.ZIP › Supplementary file/miRDeep2 file.zip!/miRDeep2 file/Know_Structure/LP42A1/oar-mir-496.pdf]

miRBase precursor : oar-mir-496
Total read count : 10
oar-miR-496-5p read count : 4
oar-miR-496-3p read count : 6
remaining reads : 0

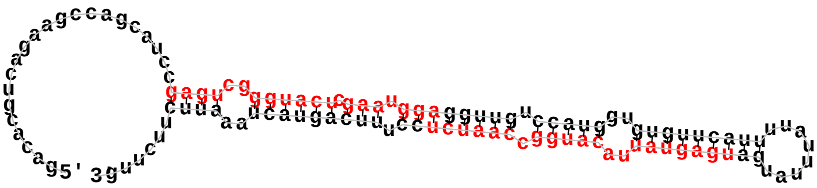

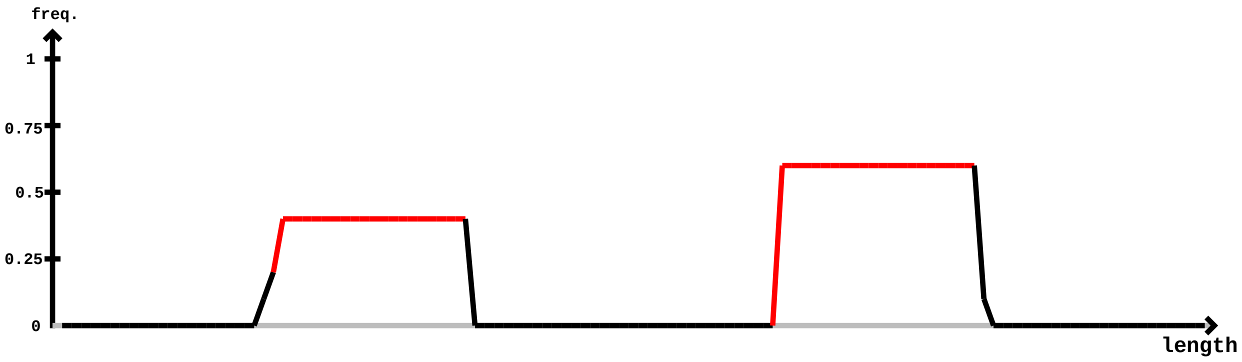


```
                                                                             oar-miR-496-3p
                        oar-miR-496-5p
5'- gacacgucagaagccagcauccgagucggguacucgaauggagguuguccaugguguguucauuuuauuuaugaugaguauuacauggccaaucuccuuucaguacuaaauucuucuug   -3'    exp
    ......................((((..((((((.(((.((((((((.(((((..(((((((((........)))))))))..))))).)))))))).)))))))))..))))......    reads      mm         sam
    ....................ccgagucggguacucgaaugga.............................................................................    1          0          sec
    .....................cgagucggguacucgaaugga.............................................................................    1          0          sec
    ......................gagucggguacucgaaugga.............................................................................    2          0          sec
    ..........................................................................ugaguauuacauggccaaucu........................    5          0          sec
    ..........................................................................ugaguauuacauggccaaucuc.......................    1          0          sec
```
